# The Role of SPEN Mutations as Predictive Biomarkers for Immunotherapy Response in Colorectal Cancer: Insights from a Retrospective Cohort Analysis

**DOI:** 10.3390/jpm14020131

**Published:** 2024-01-23

**Authors:** Yuanmei Dong, Sisi Ye, Huizi Li, Juan Li, Rongrui Liu, Yanyun Zhu

**Affiliations:** 1 Department of Medical Oncology, The Fifth Medical Center of People’s Liberation Army General Hospital, Beijing 100853, China; dongyuaner@163.com (Y.D.); hxzbb1983@163.com (S.Y.); juanli_301@163.com (J.L.); liurongrui@hotmail.com (R.L.); 2 Department of Nutrition, PLA Rocket Force Characteristic Medical Center, Beijing 100088, China; lihuiziyy@126.com

**Keywords:** colorectal cancer, immune checkpoint inhibitors, SPEN, tumor microenvironment, immunogenicity

## Abstract

Background: Colorectal cancer (CRC) is the leading cause of cancer deaths, and treatment, especially in the metastatic stage, is challenging. Immune checkpoint inhibitors (ICIs) have revolutionized CRC treatment, but response varies, emphasizing the need for effective biomarkers. This study explores SPEN mutations as potential biomarkers. Methods: Using data from the Memorial Sloan Kettering Cancer Center (MSKCC) and The Cancer Genome Atlas (TCGA)—Colorectal Cancer, this research applied bioinformatics tools and statistical analysis to SPEN (Split Ends) mutant and wild-type CRC patients treated with ICIs. Focus areas included mutation rates, immune cell infiltration, and DNA damage response pathways. Results: The SPEN mutation rate was found to be 13.8% (15/109 patients) in the MSKCC cohort and 6.65% (35/526 patients) in the TCGA cohort. Our findings indicate that CRC patients with SPEN mutations had a longer median overall survival (OS) than the wild-type group. These patients also had higher tumor mutational burden (TMB), microsatellite instability (MSI) scores, and programmed death-ligand 1 (PD-L1) expression. SPEN mutants also exhibited increased DNA damage response (DDR) pathway mutations and a greater presence of activated immune cells, like M1 macrophages and CD8+ T cells, while wild-type patients had more resting/suppressive immune cells. Furthermore, distinct mutation patterns, notably with TP53, indicated a unique molecular subtype in SPEN-mutated CRC. Conclusions: We conclude that SPEN mutations might improve ICI efficacy in CRC due to increased immunogenicity and an inflammatory tumor microenvironment. SPEN mutations could be predictive biomarkers for ICI responsiveness, underscoring their value in personalized therapy and highlighting the importance of genomic data in clinical decisions. This research lays the groundwork for future precision oncology studies.

## 1. Introduction

Colorectal cancer (CRC) is the leading cause of cancer-related death worldwide, the third leading cause of all cancers, and the fourth leading cause of mortality [1]. Its impact is felt across different populations, with differential incidence being influenced by factors such as dietary habits, lifestyle, and genetic predisposition. Despite advances in screening and early detection, a significant number of patients present with advanced or metastatic disease at diagnosis, posing a significant therapeutic and prognostic challenge [2].

The most common treatment model for CRC includes a combination of surgery, chemotherapy, and radiotherapy [3]. In early disease, surgical resection remains the cornerstone of treatment, providing a cure. However, the prognosis for metastatic CRC (mCRC) is less promising. Chemotherapy, usually in combination with targeted therapies such as anti-VEGF agents or anti-EGFR agents, is the mainstay of treatment for mCRC [4], but these approaches are limited by variable response rates, high toxicity, and the inevitable development of resistance.

The introduction of immunotherapy, particularly immune checkpoint inhibitors (ICIs), has revolutionized the treatment landscape for several cancers, including CRC. ICIs target immune checkpoint proteins like cytotoxic T-lymphocyte-associated protein 4 (CTLA-4), programmed cell death-1 (PD-1), and programmed cell death-ligand 1 (PD-L1), which cancer cells exploit to evade immune detection. By inhibiting these checkpoints, ICIs reinvigorate the immune system to recognize and attack cancer cells. In CRC, particularly for subsets of patients with specific biomarkers such as microsatellite instability-high (MSI-H) or mismatch repair deficiency (dMMR), ICIs have demonstrated substantial efficacy. These biomarkers are indicative of a high neoantigen load, which correlates with an increased likelihood of response to immunotherapy. However, the overall percentage of CRC patients who benefit from ICIs is still limited, underscoring the need for more precise predictive biomarkers [5,6,7].

Currently, several biomarkers have been employed to predict the efficacy of ICIs in CRC, including MSI-H, dMMR, tumor mutational burden (TMB), and PD-L1 expression. The objective response rates (ORRs), which are defined as the percentage of patients who achieve a response, for patients with these biomarkers are promising, ranging between 40 and 50%, yet many patients do not respond as anticipated [8]. Moreover, mutations in polymerase epsilon (POLE) or polymerase delta 1 (POLD1) have been ascertained as negative predictive markers for ICIs across diverse cancer types [9]. The immunoscore, which quantifies the number of tumor-infiltrating lymphocytes (TILs) and immune cell populations, has been proposed to hold a predictive value for immunotherapeutic response. The ongoing POCHI trial (NCT04262687), centering on microsatellite stable (MSS) metastatic CRC (mCRC) with pronounced immune infiltration as assessed by immunoscore, exemplifies the continuous effort in refining the predictive accuracy of immunotherapy responses. However, biomarkers with more predictive ability are still needed.

The SPEN (Split Ends) is a human gene that encodes the SPEN protein, which is also known as the SMRT/HDAC1-associated repressor protein (SHARP). SPEN is a nuclear-based protein integral to X-linked gene silencing and transcriptional control. This very entity has been identified as pivotal in controlling embryonic growth and subsequent development by participating in the Notch signaling pathway. Furthermore, an association between SPEN’s function and multiple cancer-related pathways—including WNT signaling plus NOTCH circuits— both crucial to the carcinogenesis of CRC, has been established [10,11]. The WNT signaling pathway is a complex network of protein interactions that functions most commonly in embryonic development and cancer but is also involved in normal physiological processes in adults. The canonical WNT signaling pathway (also known as the WNT/β-catenin signaling pathway) is a recognized driver of colon cancer and one of the most representative signaling pathways [12]. As a functional effector molecule of WNT signaling, the modification and degradation of β-catenin are key events in the WNT signaling pathway and the development and progression of colon cancer. Therefore, the WNT signaling pathway plays an important role in the pathogenesis of diseases, especially the pathogenesis of CRC [12]. The NOTCH signaling pathway is another critical mediator of tissue homeostasis and repair and is frequently co-opted during tumor development. Almost all CRCs demonstrate hyperactivation of the NOTCH pathway, which in many cases is believed to be the initiating and driving event [13]. The proper function of the NOTCH pathway is essential for normal cell development, differentiation, proliferation, and apoptosis [11].

Primarily engaged in X chromosome silencing (XCI), SPEN has a critical function in tumorigenesis and gender disparities within cancer. Through both bioinformatics analysis and immunohistochemical staining, it has been substantiated that there is indeed significant variation in SPEN expression across diverse forms of cancers. It may potentially be implicated with RNA splicing along with processing as suggested by comprehensive enrichment analysis [14,15].

Alongside its potential involvement in WNT and NOTCH signaling systems, SPEN might also contribute significantly to the pathways that handle DNA damage response (DDR). These DDR mechanisms are essential for preserving genomic stability and obstructing the build-up of mutations potentially leading to oncogenesis. Seven operational signal transduction paths form part of this DDR mechanism: Homologous Recombination Repair (HRR), Mismatch Repair (MMR), Base Excision Repair (BER), Nucleotide Excision Repair (NER), Non-homologous End Joining (NHEJ), Checkpoint Factors (CPFs), as well as Fanconi Anemia (FE) [16].

Disruptions within DDR pathways can result in accumulated genetic damage and genomic instability, which often precipitates the development of cancer. When malignancy is present, DDR typically correlates with an increased survival rate for tumor cells and a propensity toward treatment resistance. Upon exposure to platinum-induced DNA harm, cellular mechanisms trigger diverse signal transduction pathways that either stimulate cell cycle arrest—aiding DNA repair—or initiate apoptosis, leading to eventual cellular demise. This underscores the critical role played by DDR processes when counteracting chemotherapy [17,18,19]. However, the role of SPEN in these DDR pathways is not yet fully understood. However, given its involvement in the WNT and NOTCH pathways, it is plausible that SPEN could also influence DDR pathways in CRC.

Our research aims to deepen the understanding of SPEN’s multifaceted roles in CRC and evaluate its potential as a biomarker for personalized treatment strategies, particularly in the context of immunotherapy. By elucidating the relationship between SPEN mutations and CRC progression, as well as treatment response, we hope to contribute to the advancement of personalized medicine in oncology.

## 2. Materials and Methods

### 2.1. Clinical Cohorts

We sourced data from two comprehensive databases: the Memorial Sloan Kettering Cancer Center (MSKCC) dataset and The Cancer Genome Atlas—Colorectal Cancer (TCGA-CRC) dataset. The MSKCC dataset, obtained from the Bioportal website [20], encompassed clinical, survival, and genomic data, serving as our discovery dataset. In this study, we included 109 cases in the MSKCC dataset and 526 cases in the TCGA dataset. This dataset, featured in Samstein et al. [21], included CRC patients treated with ICIs and was derived from MSK-IMPACT panel sequencing. The TCGA-CRC dataset, utilized for validation, was accessed from its official portal and included genomic variation, copy number, gene expression, and clinical information. The somatic mutation data from the TCGA dataset were generated using whole-exome sequencing (WES). These comprehensive databases were fundamental in analyzing the clinical indicators associated with the SPEN mutation, as shown in Table 1, which compares the prevalence of SPEN mutations across various patient demographics and cancer stages.

We utilized institutional pharmacy records to identify patients who received at least one dose of immunotherapy, which included drugs such as atezolizumab, avelumab, durvalumab, ipilimumab, nivolumab, pembrolizumab, or tremelimumab. These patients were then cross-referenced with those who underwent MSK-IMPACT testing as part of routine clinical care. We focused our analysis on cancer types that had more than 35 patients in the initial dataset.

The majority of patients who underwent MSK-IMPACT testing on tumor tissue were enrolled in an institutional IRB-approved research protocol (NCT01775072 at ClinicalTrials.gov, accessed on 20 October 2024), with the remainder receiving testing as part of standard clinical care. All patients provided informed consent, allowing for the return of results from sequencing analyses and further research on banked specimens.

The process of tissue processing, next-generation sequencing, and analysis has been detailed previously. Notably, concurrent sequencing of germline DNA from peripheral blood was conducted for all samples to differentiate somatic tumor mutations. Patients participating in ongoing clinical trials that restricted the publication of outcome data were excluded, along with a small group of patients with localized disease treated in the neoadjuvant setting (*n* = 9) or who had localized disease. Other preceding or concurrent non-ICI treatments were not documented or considered in the analysis. Furthermore, the timing of tissue pathology on which MSK-IMPACT was performed in relation to ICI administration varied, with a minor portion of patients having testing conducted after ICI treatment.

### 2.2. Bioinformatics Analysis

We employed Fisher’s exact test and the R package ‘GenVisR’ [22] to evaluate co-mutation and exclusive mutation relationships. Mutated pathways were identified based on non-synonymous mutations. Differences in mutation rates between the SPEN mutant and wild-type samples were assessed using Fisher’s exact test. The CIBERSORT algorithm [23] was applied to TCGA dataset transcriptomic data for immune cell infiltration analysis, and the Student’s *t*-test was used to compare immune cell profiles between SPEN mutant and wild-type samples. Gene Set Enrichment Analysis (GSEA) was conducted with specified parameters using Java software: gene set database c2.cp.kegg.v7.5.1.symbols.gmt, with a minimum exclusion set of 20 (https://www.gsea-msigdb.org/gsea/index.jsp, accessed on 20 October 2024). The DNA damage response (DDR) pathway gene set from the MSigDB database was used to identify major DDR-related pathways.

### 2.3. Statistical Analyses

Survival differences and Cox multivariate analyses were performed using the R package ‘survival’. Continuous values were compared using the Student’s *t*-test or Wilcoxon rank-sum test, depending on variance homogeneity. Categorical variables were analyzed using Fisher’s exact test. All statistical tests were two-sided, with a significance threshold set at *p* < 0.05. Statistical analyses were executed on the R platform (v4.1.2).

## 3. Results

Analysis of clinical indicators associated with the SPEN mutation revealed that the mutation rate of SPEN in the MSKCC cohort was 13.8% (15 out of 109 patients), and those in the TCGA cohort had a mutation rate of 6.65% (35 out of 526 patients). Further details from the cohorts show that the prevalence of SPEN mutations does not significantly differ by age or gender. However, a significant association was found with cancer stage in the TCGA cohort, particularly at stage II, indicating a potential relationship between SPEN mutation status and cancer progression (Table 1).

**Figure 1:**
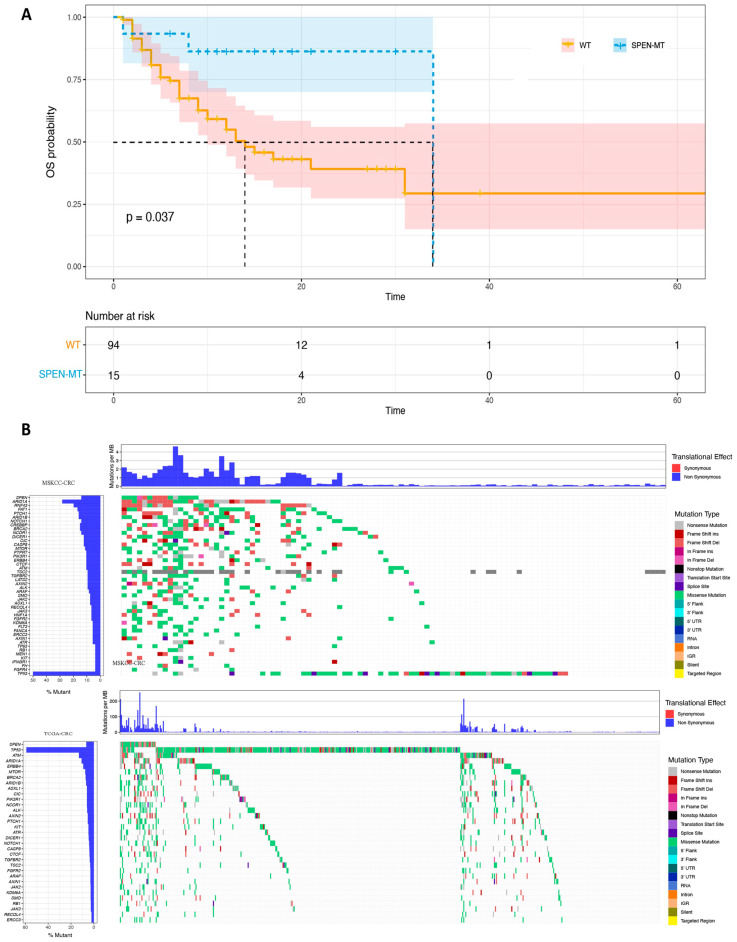
Prognostic implications and mutational patterns in SPEN mutant and wild-type CRC patients. (**A**) Kaplan–Meier survival analysis: depicts the overall survival (OS) curves for SPEN mutant and wild-type (WT) patients in the MSKCC cohort. A notable separation between the curves demonstrates a longer median OS in the SPEN mutant group, with a *p*-value of 0.037, indicating the prognostic significance of SPEN mutations. The lower chart details the “Number at risk” over time, showing a decreasing number of patients due to events or censoring. (**B**) Co-mutation landscape: illustrates the mutational landscape in the MSKCC cohort, with a focus on the frequency and exclusivity of mutations co-occurring with SPEN. TP53 is highlighted as a gene exclusively mutated in SPEN-mutated cancers, indicating potential pathways or interactions unique to this mutation profile. The comparison with the TCGA cohort validates the mutation pattern, with a significant overlap of co-mutation genes, suggesting a consistent molecular pathology across both cohorts.

The Kaplan–Meier survival plot (Figure 1A) showed a clear separation between the survival curves of SPEN mutant and wild-type (WT) patients, with a longer median overall survival (OS) in the SPEN mutant (SPEN MT) group. This underscores the prognostic significance of the SPEN mutation, highlighted by a notable *p*-value of 0.037. However, in a multivariate context, the SPEN mutation did not retain its prognostic power (*p* = 0.06), suggesting that other factors also contribute to patient survival.

The analysis of the “Number at Risk” (as shown in the lower chart of Figure 1A) provided insights into the number of individuals susceptible to events such as death or recurrence at various intervals during the study. These time points, marking intervals for data assessment, showed a decreasing trend in the number of patients “at risk” over time, attributable to events or censoring. For example, at the outset (time 0), there were 94 patients with wild type (WT) and 15 with SPEN mutations. This number gradually declined, reflecting those who had not yet experienced the event and remained under observation. The survival curve in Figure 1A enables us to deduce the survival probability at different times for each group. The *p*-value of 0.037 signifies a statistically significant difference in survival rates between the WT and SPEN mutant groups.

Furthermore, the study investigated a correlation between SPEN mutations and clinical characteristics, uncovering a significant link with the primary stage of cancer as classified by the AJCC. A higher proportion of patients with SPEN mutations were diagnosed at an earlier stage of the disease, predominantly stage II.

In the mutational landscape depicted in Figure 1B, the study also examined other genetic markers for their mutation frequency and potential co-occurrence or exclusivity with SPEN mutations. This led to the identification of 44 genes that frequently co-mutate with SPEN. Notably, TP53 was identified as a gene that mutates exclusively in association with SPEN mutations, suggesting a potential unique interaction or pathway in SPEN-mutated cancers.

The mutational profile from the TCGA cohort was used as a comparison to the MSKCC data. A substantial portion of these co-mutation genes, 72.1%, were also observed in the TCGA cohort, validating the findings across different populations. The significant overlap of co-mutation genes between the two cohorts suggests a shared molecular pathology, a consistent mutation pattern observed in our study between the MSKCC and TCGA datasets.

**Figure 2:**
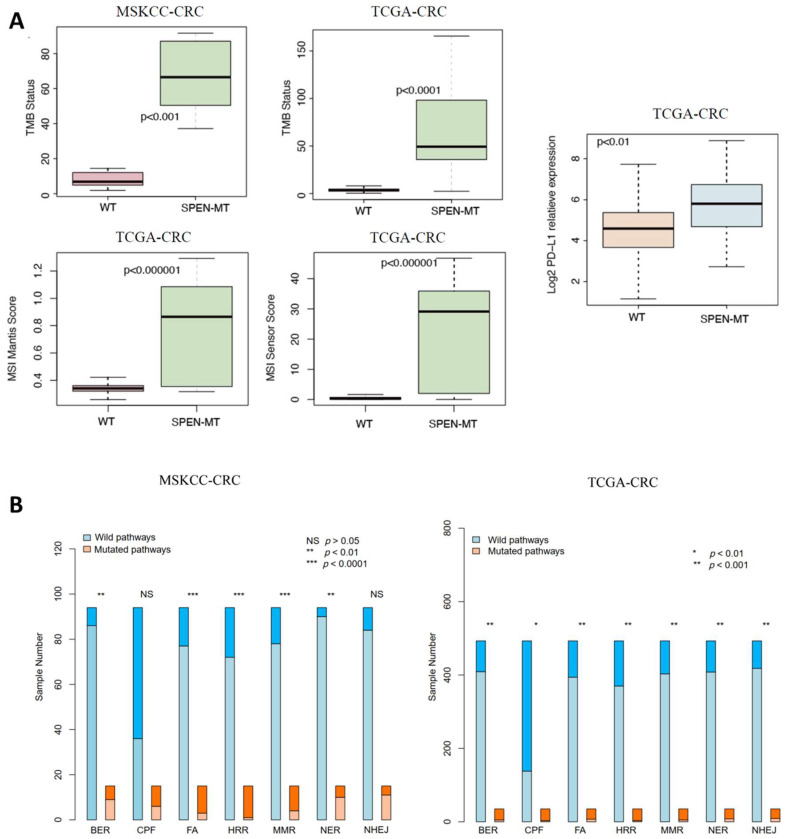
Comprehensive genomic analysis in SPEN mutant and wild-type CRC patients. (**A**) Tumor mutational burden (TMB), microsatellite instability (MSI) scores, and PD-L1 expression: comparison between SPEN mutant and wild-type (WT) groups in the MSKCC and TCGA cohorts. Elevated TMB, MSI scores, and PD-L1 expression levels were observed in the SPEN mutant group, indicating enhanced immunogenicity. (**B**) DNA damage response (DDR) pathway mutations: analysis of mutation counts in key DDR pathways including Base Excision Repair (BER), Fanconi Anemia (FA), Homologous Recombination Repair (HRR), Mismatch Repair (MMR), Nucleotide Excision Repair (NER), and Non-homologous End Joining (NHEJ). The SPEN mutant group in the MSKCC cohort shows significant mutation counts in BER, FA, HRR, MMR, and NER, while in the TCGA cohort, this extends to NHEJ.

This complex mutational interplay, including the frequency and type of mutations across various genes, offers insights into the genomic landscape that may influence the response to ICI treatment in CRC patients. Such comprehensive genomic profiling is crucial for advancing personalized medicine, enabling clinicians to tailor treatments based on individual mutational backgrounds. The analysis also demonstrated a significant increase in TMB among SPEN mutant patients in both the MSKCC and TCGA cohorts (*p* < 0.01 for both; Figure 2A), suggesting a potential for a higher neoantigen load and greater immunogenicity. The MANTIS score, indicative of microsatellite instability (MSI), was significantly elevated in the SPEN mutant group of the TCGA cohort (*p* < 0.01; Figure 2A), pointing to a heightened state of genomic instability, often associated with a better response to ICIs. Additionally, the mutant group showed significantly higher MSI sensor scores (*p* < 0.01; Figure 2A) and elevated PD-L1 (CD274) expression levels (*p* < 0.01; Figure 2A) compared to the WT group.

A detailed investigation into the DDR pathways revealed a substantially higher mutation count in critical DDR pathways, including Base Excision Repair (BER), Fanconi Anemia (FA), Homologous Recombination Repair (HRR), Mismatch Repair (MMR), and Nucleotide Excision Repair (NER) in the MSKCC SPEN mutant cohort (*p* < 0.05). In the TCGA cohort, this increase extended to the Non-homologous End Joining (NHEJ) pathway as well (*p* < 0.05; Figure 2B), indicating a widespread impairment in DNA repair mechanisms in SPEN mutant tumors.

**Figure 3:**
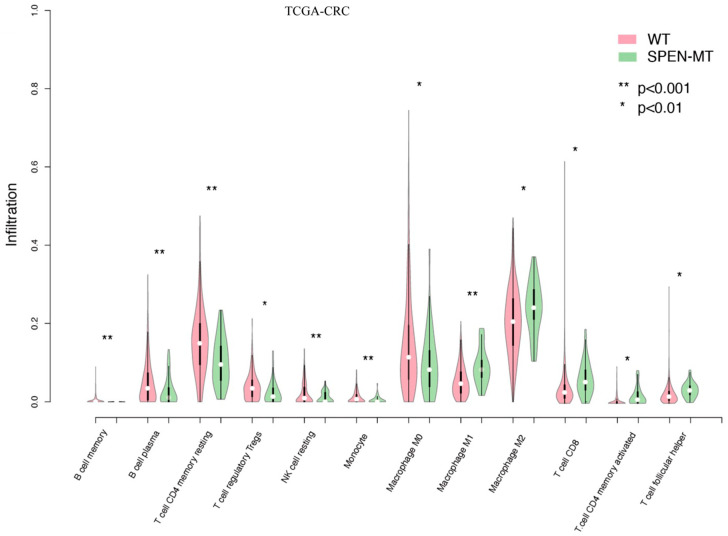
Immune cell infiltration in SPEN mutant and wild-type CRC patients using CIBERSORT: delineation of immune cell abundance in the TCGA-CRC cohort. The WT group exhibits higher levels of resting/suppressive immune cells, whereas the SPEN mutant group demonstrates a significant increase in activated immune cells, indicative of an inflammatory tumor microenvironment.

The CIBERSORT algorithm estimated the abundance of immune cells in CRC patients, revealing a distinct immune landscape in SPEN mutant tumors. In the TCGA-CRC cohort, the WT group exhibited higher levels of resting/suppressive immune cells, such as resting memory CD4+ T cells, resting NK cells, and regulatory T cells (all *p* < 0.01; Figure 3). Conversely, the SPEN mutant group displayed significantly increased proportions of activated immune cells, including M1 macrophages, activated memory CD4+ T cells, and CD8+ T cells (all *p* < 0.01; Figure 3). This suggests that SPEN mutations are associated with an inflammatory tumor microenvironment (TME), conducive to ICI therapy.

**Figure 4:**
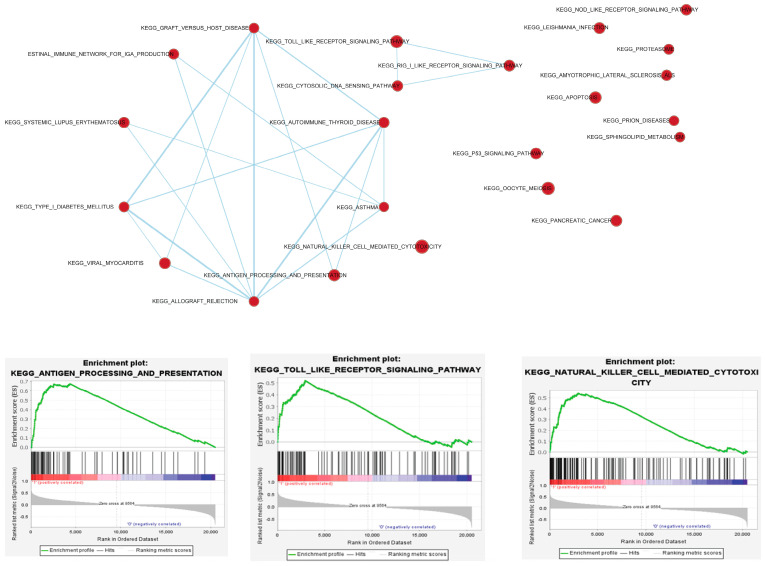
Gene Set Enrichment Analysis (GSEA) highlighting immune pathway activation in SPEN mutant CRC patients. This figure displays the results of the GSEA, demonstrating significant enrichment of immune-related pathways in the SPEN mutant group compared to the wild-type (WT) group in CRC patients. Key pathways such as antigen processing and presentation, natural killer cell-mediated cytotoxicity, and toll-like receptor signaling show heightened activity in the mutant group, as indicated by enrichment scores greater than 0. The enrichment of these pathways, with a significance level of *p* < 0.05, suggests an enhanced potential for immune response activation, which may contribute to the effectiveness of ICIs in these patients.

The Gene Set Enrichment Analysis (GSEA) plots (Figure 4) indicated significant enrichment of immune-related pathways in the SPEN mutant group. Key pathways such as antigen processing and presentation, natural killer cell-mediated cytotoxicity, and toll-like receptor signaling were more active in the mutant group compared to the WT group, as suggested by an enrichment score greater than 0. The significance level for this enrichment is *p* < 0.05, as depicted in the enrichment plots. This activation of immune-related pathways in the mutant group could potentially enhance the effectiveness of ICIs.

In summary, SPEN mutations could serve as predictive biomarkers for ICI responsiveness and may guide the development of personalized therapeutic approaches in CRC.

## 4. Discussion

Our study sheds light on the ramifications of SPEN mutations in colorectal cancer, especially in relation to ICI therapy. This finding is crucial given the current challenges that come with managing CRC, particularly during its metastatic stage. There has been a significant improvement over time in therapeutic approaches for CRC due to advancements in surgical techniques, the implementation of targeted therapies, and more recently, the advent of immunotherapies. Nevertheless, the responses of patients to these therapies display substantial inconsistency, underscoring the necessity for dependable predictive biomarkers that can guide therapeutic decisions. Our study identifies changes within SPEN as potential indicators of susceptibility towards ICIs which could significantly impact patient classification and improve healthcare outcomes.

To better understand the predictive importance of SPEN mutations in CRC, it is helpful to compare them with established biomarkers. For example, mismatch repair deficiency (dMMR) and high microsatellite instability (MSI-H) have been instrumental in distinguishing patients who are probable beneficiaries of ICIs. Research indicates that CRC patients exhibiting dMMR/MSI-H show greater response rates to ICIs compared to those demonstrating proficiency in mismatch repair (pMMR)/microsatellite stability (MSS) [24,25]. The potential for SPEN mutations to fulfill a similar function, particularly given their correlation with an elevated TMB, necessitates further investigation. A study conducted by Le et al. demonstrated that patients afflicted with dMMR CRC displayed significantly extended progression-free survival and overall survival when administered pembrolizumab, as an ICI, compared to traditional chemotherapy [26]. Our findings suggest that, in a similar manner, SPEN mutations might classify patients who are likely to benefit from ICIs.

SPEN mutations’ influence extends beyond the realm of CRCs, as evident through its participation in various cancer-related pathways observed within other malignancies. In breast cancer cases, it has been suggested that SPEN functions as a regulator for NOTCH and WNT signaling pathways [27], both crucially involved during tumorigenesis and metastasis processes. This multifaceted nature associated with SPEN hints at intricate interactions involving cellular pathways which may provide therapeutic benefits if leveraged appropriately. As seen in hepatocellular carcinoma instances where alterations concerning the WNT/β-catenin pathway were linked to resistance against immunotherapy [28], this suggests possible links between treatment outcomes related to CRCs and specific genetic changes such as those identified within our studies.

Our study also highlighted co-mutation patterns, specifically exclusivity regarding TP53 gene variations, opening up fresh perspectives toward understanding pathogenesis-related intricacies inherent in colorectal cancers. This observation mirrors patterns seen within other malignancies where specific mutation sequences have been linked to unique clinical behaviors and responses toward treatment. For example, in ovarian cancer cases, BRCA1/2 mutations were associated with positive response rates toward platinum-based chemotherapy alongside PARP inhibitors [29,30]. Similarly, concerning lung cancer instances, EGFR gene alterations dictate the selection of tyrosine kinase inhibitors [31]. The exclusivity observed between SPEN and TP53 mutations could represent a distinctive molecular subtype that responds differently to ICIs, thereby underlining the necessity for personalized treatment strategies. The altered immune landscape characterized by an influx of activated immune cells present within CRCs exhibiting SPEN mutations is particularly noteworthy. These findings align perfectly with recent studies underscoring tumor microenvironments’ crucial role when determining immunotherapy efficacy levels. Studies conducted on melanoma and renal cell carcinoma revealed that pre-existing inflamed tumor environments rich in T cell infiltrations are indicative of better ICI response rates [32,33,34]. Our study suggests that these identified genetic changes might serve as markers indicating an active immunological environment inherent in CRC.

Furthermore, the increased TMB noticed among tumors showing SPEN-related variations also necessitates additional investigation. Higher than normal TMB values correlate directly with elevated neoantigen loads which enhance overall tumor visibility, making them more susceptible to attacks from our body’s own defense mechanisms. A research paper published by Aggarwal et al. suggested that high TMB was correlated positively with improved survival rates among patients suffering from various forms of cancer who underwent immunotherapies [35]. This correlation between TMB and ICI response in our study implies not only predictive capabilities regarding therapy effectiveness but also provides insights into underlying biological factors governing patient responses to such therapies.

The translation of the findings of this study into clinical application presents few obstacles. The initial stage entails corroborating these findings in larger and more diverse groups of patients. Furthermore, it is imperative to conduct functional investigations that elucidate the mechanistic connections between SPEN mutations and the immune response in CRC. Such inquiries hold promise for providing invaluable insights into how these mutations impact tumor–immune interactions as well as the effectiveness of immunotherapy.

Our investigation also underscores the significance of incorporating genomic data into clinical decision-making processes. As precision oncology progresses, tailoring treatments based on individual genetic profiles will assume increasing importance. In this context, integrating SPEN mutations into multi-gene panels utilized for CRC prognostication and therapy selection could prove advantageous.

## 5. Conclusions

In conclusion, our study offers new insights into CRC, especially in the context of immunotherapy. The potential of SPEN mutations as biomarkers for guiding ICI therapy aligns with the broader goal of personalizing CRC treatment. While our findings suggest that SPEN mutations could be instrumental in selecting and optimizing ICI therapy, thereby enhancing treatment strategies, it is important to acknowledge the limitations of our study, particularly its retrospective nature. These results contribute to the growing field of cancer genomics and set the stage for future research, which is essential for refining the precision and effectiveness of cancer treatments. Further investigations, ideally with prospective designs, are necessary to fully validate and understand the implications of SPEN mutations in CRC therapy.

## Figures and Tables

**Table 1:** Clinical indicators associated with the SPEN mutation.

Categories		MSKCC SPEN WT	MSKCC SPEN MT	*p*-Value	TCGA SPEN WT	TCGA SPEN MT	*p*-Value
Age	<60	62	9	0.74	169	7	0.76
	>60	32	6		322	28	
Gender	Female	43	5	0.41	235	18	0.1
	Male	51	10		256	17	
Stage	I				88	6	0.049
	II				177	21	
	III				146	5	
	IV				69	2	

SPEN WT: SPEN wild-type, indicating the patients whose SPEN gene did not have mutations. SPEN MT: SPEN mutant, referring to the patients with mutations in their SPEN gene.

## Data Availability

The data that support the findings of this study are available from the corresponding author upon reasonable request.
